# Inhibiting DNA methylation improves antitumor immunity in ovarian cancer

**DOI:** 10.1172/JCI160186

**Published:** 2022-07-15

**Authors:** Katherine B. Chiappinelli, Stephen B. Baylin

**Affiliations:** 1 Department of Microbiology, Immunology, and Tropical Medicine and; 2 GW Cancer Center, The George Washington University, Washington, DC, USA.; 3 Johns Hopkins University School of Medicine and the Sidney Kimmel Comprehensive Cancer Center, Baltimore, Maryland, USA.; 4 Van Andel Institute, Grand Rapids, Michigan, USA.

## Abstract

Cancer cells resist the immune response in a process known as immune editing or immune evasion. Therapies that target the immune system have revolutionized cancer treatment; however, immunotherapies have been ineffective for the majority of ovarian cancer cases. In this issue of the *JCI*, Chen, Xie, et al. hypothesized that hypomethylating agent (HMA) treatment would induce antitumor immunity to sensitize patients with ovarian cancer to anti-PD-1 immunotherapy. The authors performed a phase II clinical trial to test the combination of guadecitabine, a second-generation HMA, along with pembrolizumab, an immune checkpoint inhibitor of PD-1. The trial included a group of 35 patients with platinum-resistant ovarian cancer. While the clinical benefit from the combined HMA plus immune checkpoint blockade regimen was lower than hoped, the correlate analyses gave important information about which patients with ovarian cancer may be more likely to respond to immune therapy.

## Advances in cancer therapies reverse immune evasion

Cancer cells resist the immune response in a process known as immune editing or immune evasion. Initially, immune cells — including natural killer cells and T effector cells — fight and kill cancer cells. As cancers progress, however, malignant cells exhibit mechanisms of immune suppression, known as immune tolerance. In this latter state, tumors often express PD-L1, a ligand for the PD-1 protein on CD3^+^ T cells, that inhibits killer T cell action against tumor cells. T regulatory cells that express CTLA-4 secrete cytokines to inhibit the action of T and natural killer cells against tumors (1). Recent therapies that reverse this evasion, specifically via the inhibition of PD-1 and CTLA-4, termed “immune checkpoint blockade,” have shown durable responses for a portion of patients with solid tumors and are especially effective in melanoma and non**–**small cell lung cancer. While tumor-infiltrating T cells predict better prognosis in ovarian cancer (2, 3), thus far, immune checkpoint blockade has not produced durable responses (4). A recent trial compared (a) the anti-PD-1 agent nivolumab alone with (b) nivolumab plus the anti-CTLA-4 agent ipilimumab in 100 patients with recurrent or persistent ovarian cancer. The trial demonstrated a 12% response rate in the nivolumab group and a 31% response rate in the combination group. However, the increase in progression-free survival was minimal (4).

## Combination epigenetic and immune therapy

In this issue of the *JCI*, Chen et al. sought to combine an epigenetic modulator, specifically, a hypomethylating agent (HMA), with the anti-PD-1 agent pembrolizumab to increase immune signaling in ovarian cancer and improve response to immune checkpoint blockade (5). A substantial body of preclinical data shows that HMAs — which inhibit the DNA methyltransferase (DNMT) enzymes that add the silencing DNA methylation mark to DNA — increase immune signaling in cancer cells. HMAs boost immune signaling in tumors through activation of type I and III interferon signaling, which is induced following the detection of double-stranded RNA, including DNA–methylated transposable elements (TEs) (6–8). Low doses of the HMAs azacytidine (Aza) (9) and 5-aza-2′-deoxycytidine (10) upregulate immune signaling, including the interferon response, cytokines, and antigen processing and presentation in breast, colon, lung, and ovarian cancer cell lines (6–8, 11). HMAs activate a canonical interferon signaling pathway through upregulation of dsRNA, reducing global DNA methylation and inducing viral mimicry through the expression of TEs that activate dsRNA sensors (7). In addition, HMAs have recently been shown to activate inflammasome signaling, an antipathogen pathway that includes viral mimicry as a component (12). Treatment with HMAs along with another epigenetic modifier, histone deacetylase inhibitors (HDACis), increased TEs in a mouse model of ovarian cancer, activating interferon signaling and recruiting CD8^+^ T cells to kill the tumors and sensitize this model to anti-PD-1 therapy (13). Similarly, in a murine model of non**–**small lung cancer, the combination of HDACi with HMA showed comparable effects, linking enhanced tumor-immune signaling with downregulation of the oncogene CMYC and its target genes, reversing immune exhaustion in CD8^+^ T cells (14). Two other preclinical studies showed that combining the HMA decitabine with anti-CTLA-4 (15) or azacytidine with anti-PD-L1 (16) sensitized murine ovarian cancers to immune checkpoint blockade therapy by increasing interferon and chemokine signaling from the tumor cells to recruit and activate host immune cells. Translating these findings to the clinic, a recent phase Ib trial that combined HMA treatment with anti-CTLA-4 in patients with melanoma showed promising results, including improved immune activation and antitumor activity (17). This therapeutic combination is currently being tested in clinical trials for melanoma, colorectal cancer, ovarian cancer, and kidney cancer, among others (17, 18). In this ovarian cancer trial, Chen, Xie, et al. administered the HMA guadecitabine on days 1–4 at 30 mg/m^2^, followed by 200 mg pembrolizumab on day 5, with this regimen repeated every 21 days. In 35 evaluable patients, 3 patients had partial responses (8.6%) and 8 (22.9%) showed stable disease. The median duration of this clinical benefit was 6.8 months, and in general, the combination was well tolerated. Median progression-free survival was 1.7 months with a median overall survival of 16.3 months. Unfortunately, this combination did not provide enough clinical benefit to advance the regimen for further drug development.

Comparing patient responses from the trial performed by Chen, Xie, et al. with other trials in the epigenetic and immune therapy space shows some benefit from this combination. The responses in Chen, Xie, et al. compare favorably with the METADUR trial, which combined the anti-PD-1 agent durvalumab with an oral HMA azacytidine (also called CC-486) in advanced solid tumors. Patients in the METADUR study exhibited no objective or partial responses and had a stable disease rate of 7.1%, with a median progression-free survival of 1.9 months and a median overall survival of 5 months (19). However, the combination of 2 immune checkpoint blockade agents (the anti-PD-1 nivolumab and the anti-CTLA-4 ipilimumab) in 100 patients with persistent or recurrent ovarian cancer was superior to the guadecitabine/pembrolizumab combination, with 12% response rate in the nivolumab group and 31% response rate in the combination group. Similar to the Chen, Xie, et al. trial, these responses were not durable, with median progression free survival of 2 months in the nivolumab group and 3.9 months in the nivolumab plus ipilimumab group (4).

## Predicting response to immune therapy

Importantly, Chen, Xie, et al. obtained tumor biopsies, PBMCs, and plasma, both before and after treatment. Correlative data from these patients provide information about the tumor microenvironment in ovarian cancers and which patients might respond to immune therapy or derive benefit from a combination of immune and epigenetic therapies. Notably, analyses of posttreatment versus pretreatment biopsies revealed transcriptional upregulation of immune signaling, and CyTOF analysis showed that patients with durable clinical benefit — defined as patients with partial response or stable disease — tended to have more naive CD4^+^ cells and specific monocyte populations. In addition, patients with clinical benefit had more immune cells, specifically CD8^+^ T cells and CD20^+^ B cells, touching tumor cells in biopsies compared with non-responders. CD8^+^ T cell proximity to tumor cells has previously been shown to predict response to immune checkpoint blockade in melanoma (20). In addition, the patients with durable clinical benefit had more classical monocytes and dendritic cells, while nonresponders had more non-classical monocytes prior to therapy. Nonresponders exhibited higher levels of PD-1 and PD-L1 on their monocytes and dendritic cells, suggesting an important role for the myeloid compartment in immune suppression in this disease. Multiplex immunohistochemistry analysis of tumor biopsies revealed that patients with clinical benefit had higher CD20^+^ B cells and tertiary lymphoid structures in their tumors at baseline and posttreatment compared with non-responders. These data support recent findings in clinical trials of immune checkpoint blockade in melanoma (21) and renal cell carcinoma (22), in which the presence of B cells and tertiary lymphoid structures predicts a response to immune therapy. In addition, a recent study found that the presence of IgG in high grade serous ovarian cancer tumors positively correlates with survival (23). B cells in these ovarian cancer tumors produce autoantibodies against proteins overexpressed by ovarian cancer, including MMP14 (23). These data should prompt further mechanistic studies and potential strategies to boost B cell function in combination with immune checkpoint blockade for improved responses in ovarian cancer.

## HMA dosage

While Chen, Xie, et al. reference transcriptomic changes associated with HMA preclinically, they observed minimal changes in DNA methylation when comparing posttreatment and pretreatment biopsies. The authors observed about a 5% decrease of LINE-1 methylation in PBMCs and a small change in average β value (measuring genome-wide methylation) in tumor samples. These changes were less robust than methylation changes upon guadecitabine treatment in acute myeloid leukemia, where 15–25% demethylation of LINE1 elements in PBMCs was observed (24). It should be noted that this latter trial utilized guadecitabine at 60 or 90 mg/m^2^ for 5 or 10 days on a 28-day treatment schedule, with the most robust demethylation reported in the 10-day schedule. The lower dose of guadecitabine in the study by Chen, Xie, et al. may be responsible for the less robust LINE1 demethylation. In addition, the challenge of HMA drug delivery should not be ignored. Results from the phase II METADUR trial were recently published, providing results for the efficacy of the oral Aza CC-486 with the anti-PD-1 agent durvalumab in patients with colorectal cancer, ovarian cancer, and breast cancer (19). The researchers concluded that viral mimicry was not induced, as CC-486 did not penetrate the tumors, leading to no clinical responses (19). Chen, Xie et al. reported some increased expression of potential tumor suppressor genes, but these genes were not previously characterized by abnormal promoter CpG island hypermethylation (25). For the above reasons, optimizing HMAs for therapy in solid tumors to induce a robust immune response is a critical goal for future combinations of epigenetic and immune therapy. A recently characterized DNMT1-selective inhibitor that has improved in vivo tolerability compared with decitabine may provide opportunities for future combinations with immune therapy in solid tumors (26).

## Conclusions

While the percentage of patients (34%) that received clinical benefit from the combined HMA plus immune checkpoint blockade regimen was not enough to move forward with this therapy, the correlate analyses in Chen, Xie, et al. give important information about which patients with ovarian cancer may be more likely to respond to immune checkpoint blockade therapy. Specifically, the presence of tertiary-lymphoid structures and the number of B and CD8^+^ T cells contacting tumor cells seems to positively predict response in this malignancy, which has had low response rates to immune checkpoint blockade. Lastly, this work provides important information about dosage and scheduling of HMAs in solid tumors. Overall, the results of this study should inform future work combining epigenetic and immune checkpoint blockade therapies in platinum-resistant ovarian cancer.

## Version 1 07/15/2022

Electronic publication
